# Urinary symptoms are correlated with quality of life after deep brain stimulation in Parkinson's disease

**DOI:** 10.1002/brb3.1164

**Published:** 2018-11-19

**Authors:** Tatsuya Yamamoto, Tomoyuki Uchiyama, Masato Asahina, Yoshitaka Yamanaka, Shigeki Hirano, Yoshinori Higuchi, Satoshi Kuwabara

**Affiliations:** ^1^ Department of Neurology, Graduate School of Medicine Chiba University Chiba Japan; ^2^ Department of Neurology International University of Health and Welfare Ichikawa Japan; ^3^ Neurology Clinic Tsudanuma Japan; ^4^ Department of Neurological Surgery, Graduate School of Medicine Chiba University Chiba Japan

**Keywords:** deep brain stimulation, health‐related quality of life, Parkinson’s disease, urinary symptoms

## Abstract

**Aims:**

Deep brain stimulation (DBS) is known to dramatically improve motor complications in patients with Parkinson's disease (PD), but its effect on urinary symptoms and health‐related quality of life (HRQOL) remains unknown. We aimed to examine the relationship between urinary symptoms and HRQOL in patients with PD who underwent DBS.

**Methods:**

The International Prostate Symptom Score (IPSS) and overactive bladder symptom score (OABSS) were determined to evaluate urinary symptoms in patients with PD who underwent DBS. Postoperative evaluations were performed at 3 months, 1 year, and 3 years postoperatively. We also performed a urodynamic study (UDS) in 13 patients with PD preoperatively and postoperatively. A follow‐up UDS was performed 2.0 ± 0.5 years postoperatively.

**Results:**

The preoperative urinary symptoms questionnaire was completed by 28 patients, of whom 14 completed the postoperative urinary symptoms questionnaire after 3 months, 18 after 1 year, and 10 after 3 years. The mean OABSS and IPSS did not change significantly at any follow‐up periods postoperatively. When assessing the relationship between urinary symptoms and HRQOL and motor functions, the OABSS and IPSS showed significant positive correlations with HRQOL at 3 months postoperatively. The OABSS and IPSS showed significant positive correlations with activities of daily living (ADL) during the off‐phase at 3 years postoperatively. All urodynamic parameters remained unchanged postoperatively.

**Conclusions:**

Deep brain stimulation did not significantly affect urinary dysfunctions in patients with PD. Urinary symptoms might partially contribute to HRQOL at 3 months postoperatively and ADL during the off‐phase at 3 years postoperatively.

## 1 INTRODUCTION

Deep brain stimulation (DBS) of the subthalamic nucleus (STN) and globus pallidus interna (GPi) is a standard therapy for patients with advanced stages of Parkinson's disease (PD) with motor complications such as dyskinesia and wearing‐off (Okun, 2012). DBS improves motor complications in patients with PD. Furthermore, several non‐motor symptoms including autonomic dysfunctions, dementia, and neuropsychiatric symptoms generally occur in advanced stages of PD (Kalia and Lang, 2015). Among autonomic dysfunctions, urinary dysfunctions are severe and prevalent in patients with PD (Sakakibara, Panicker, Finazzi‐Agro, Iacovelli, & Bruschini, 2016). In particular, overactive bladder symptoms, including urinary urgency or urgent urinary incontinence, are common in advanced stages of PD (Sakakibara et al., 2016). Quality of life (QOL) in patients with PD is seriously deteriorated by the presence of urinary dysfunctions. Drug therapies, such as anticholinergics and β3 adrenoceptor agonists, are only partially effective in patients with PD (Sakakibara et al., 2016). Although DBS might be an important treatment option for urinary dysfunctions, the effect of DBS on urinary dysfunctions in patients with PD remains controversial (Finazzi‐Agrò et al., 2003; Seif et al., 2004; Winge et al., 2007; Witte et al., 2018).

We have previously reported that high‐frequency electrical stimulation of the STN significantly inhibited bladder contractions in normal cats (Sakakibara et al., 2003). We have also revealed that many of the recorded neurons in the STN preferentially fired during the bladder storage phase in normal cats (Sakakibara et al., 2003), suggesting that STN‐DBS might be effective for patients with PD with urinary dysfunctions.

Seif, et al. (2004) reported that STN‐DBS significantly increased the maximum capacity of the bladder in PD patients and concluded that STN‐DBS has a urodynamically and significant recordable effect which result in normalization of pathologically increased bladder sensibility. Finazzi‐Agrò, et al. (2003) reported that STN‐DBS increased bladder capacity and decreased the amplitude at the detrusor overactivity. Herzog, et al. (2006, 2008) demonstrated that STN‐DBS in patients with PD normalized the activity of the anterior cingulate cortex and prefrontal cortex, which are usually overactive, thereby improving lower urinary tract symptoms (urinary symptoms) in patients with PD. However, Winge, et al. (2007) reported that none of the primarily measured urodynamic parameters were significantly changed by STN‐DBS. Although several other studies also revealed that urinary symptoms, such as urinary frequency or urgency, significantly improved after DBS, the degrees of their improvement were very slight and not clinically relevant; moreover, it is unknown whether DBS significantly improves urinary dysfunctions in patients with PD (Finazzi‐Agrò et al., 2003; Seif et al., 2004; Winge et al., 2007; Witte et al., 2018). The effect of DBS on urinary symptoms in PD patients is controversial.

Furthermore, the detailed relationships between urinary symptoms and motor dysfunction and health‐related QOL (HRQOL) are also unknown, which is important for the clinical examination of patients with PD after DBS. We have previously reported that HRQOL and motor symptoms were not necessarily correlated despite the significant improvement in motor functions, suggesting that non‐motor symptoms might affect HRQOL after DBS (Yamamoto, Uchiyama, et al., 2017). Among several non‐motor symptoms, urinary symptoms are known to be prevalent and severe in PD patients (Schapira, Chaudhuri, & Jenner, 2017). However, temporal changes in urinary symptoms and their relationship to HRQOL after DBS are unknown.

The present study aimed to clarify the effect of DBS on urinary symptoms in patients with PD and examine the relationship between urinary symptoms and motor functions and HRQOL.

## 2 MATERIALS AND METHODS

### 2.1 Patient evaluation

This study enrolled 28 patients undergoing DBS implantation (STN: *n* = 22, GPi: *n* = 6) at Chiba University Hospital between December 2009 and December 2017. Patients were diagnosed with PD according to the clinical diagnostic criteria of the United Kingdom Parkinson's Disease Society Brain Bank (Gibb & Lees, 1988). All participants had reported severe motor fluctuations and complications. GPi‐DBS was selected for patients who had markedly troublesome dyskinesia before surgery. Before enrollment in this study, the participants had been treated with anti‐PD medications and had been taking L‐3,4‐dihydroxyphenylalanine, decarboxylase inhibitors, dopamine agonists, selegiline, and entacapone. The motor functions during the on‐ and off‐phases were examined using the Unified Parkinson's Disease Rating Score (UPDRS) Part I, Part II, Part III, and Part IV both preoperatively and postoperatively. HRQOL was assessed using the PD questionnaire‐39 (PDQ‐39) summary index (SI) preoperatively and postoperatively, and cognitive functions were evaluated with the Mini Mental State Examination (MMSE) and the Frontal Assessment Battery (FAB). We evaluated HRQOL using the PDQ‐39 because the PDQ‐39 is the most widely used disease‐specific, patient‐completed scale assessing PD. The PDQ‐39 comprises 39 items grouped into eight subscales, including mobility, activities of daily living (ADL), emotional well‐being, stigma, social support, cognition, communication, and bodily discomforts. A higher score on the PDQ‐39 also represents worse conditions. The PDQ‐39 SI was assessed during stimulation after DBS, and the levodopa equivalent dose (LED) of anti‐PD medication was calculated as described elsewhere (Tomlinson et al., 2010). We used overactive bladder symptom score (OABSS) and International Prostate Symptom Score (IPSS) to assess the urinary symptoms because both scales are widely used for the evaluation of urinary symptoms and are easily undertaken by patients. OABSS is used as the evaluation of storage dysfunctions (daytime and night‐time urinary frequency, urinary urgency, and urgent urinary incontinence), whereas IPSS is used as the evaluation of both storage (urinary frequency, nocturia, and urgency) and voiding dysfunctions (incomplete emptying, intermittency, weak stream, and straining). The higher scores of OABSS and IPSS represent the worse condition. The OABSS and IPSS were determined to assess urinary symptoms in patients with PD (mean age: 65.5 ± 1.6 years, mean disease duration: 11.8 ± 1.0 years) who underwent DBS. The OABSS and IPSS were determined both before and after DBS. Postoperative evaluations were performed at 3 months, 1 year, and 3 years postoperatively. The temporal changes in urinary symptoms were examined in patients with PD after undergoing DBS.

The correlation coefficients between urinary symptoms (OABSS and IPSS) and motor functions (UPDRS) and HRQOL (PDQ‐39) were calculated.

### 2.2 Urodynamic study (UDS)

Of the 28 patients, 13 agreed to be evaluated for urinary dysfunctions via a urodynamic study preoperatively and postoperatively. The urodynamic evaluation was performed between April 2012 and December 2017. A follow‐up UDS was performed at 2.0 ± 0.5 years postoperatively.

The UDS was performed by neurologists and urologist who were familiar with free‐flowmetry and urodynamic findings. The neurologists evaluated urinary dysfunction associated with neurological dysfunction in detail. The average and maximum flow rates were acquired by performing free‐flowmetry before conducting a pressure‐flow study (PFS). Postvoid residual (PVR) (normal volume <50 ml) was measured via transurethral catheterization after the voided volume was measured. Cystometry and electromyography (EMG) was conducted using a urodynamic computer (Janus; Life‐Tec, Houston, TX, USA) and an EMG computer (Neuropack Sigma; Nihon Kohden, Tokyo, Japan). During the cystometry and PFS, the EMG of the anal sphincter was recorded continuously by inserting a coaxial needle electrode into the EAS muscles. An 8‐Fr double‐lumen catheter was inserted transurethrally, and water (saline) cystometry was performed at an infusion rate of 50 ml/min while the patient remained seated. Rectal pressure was measured simultaneously with a balloon catheter. Rectal pressure was electronically subtracted from the intravesical pressure. PFS follows water cystometry. Both free‐flowmetry and PFS were carried out with the patient in the sitting position in the same environment, and PVR was measured after PFS. Tracings from each flow study were evaluated by the neurologists and the urologist.

Abnormal urodynamic findings during the voiding phase included impaired bladder contractile function. The degree of detrusor contractions was examined using Schäfer's nomogram, which categorizes detrusor contractility as strong, normal, weak, or very weak, depending on the flow rate and detrusor pressure. Abnormal urodynamic findings during the storage phase included detrusor overactivity (DO), which was defined as involuntary detrusor contractions during the filling phase. Quantitative parameters obtained during the storage phase included bladder volume at first desire to void (FDV) and strong desire to void (SDV); FDV <100 ml and SDV <300 ml values were considered abnormal findings. The maximum cystometric capacity was the volume at which the patient could no longer delay micturition. The methods, definitions, and units employed conformed to the standards recommended by the International Continence Society (Schäfer et al., 2002).

### 2.3 Ethical considerations

This study was approved by the Chiba University Hospital Institutional Review Board. All participants provided written informed consents that were obtained during their on‐time. None of the participants had a compromised capacity/ability to consent in this study.

### 2.4 Statistical analysis

All data were expressed as mean ± standard error of mean. All statistical analyses were carried out using SPSS version 22.0 (IBM, Armonk, USA). We used Dunnett's tests for comparisons between baseline and postoperative UPDRS, OABSS, IPSS, cognitive functions (MMSE and FAB), and PDQ‐39 scores at each follow‐up points. Tukey's test was used to determine which urinary symptoms among OABSS and IPSS were the most bothersome symptoms. The Spearman's rank correlation coefficient was calculated to examine the relationship between IPSS, OABSS, HRQOL (PDQ‐39 SI and subscores), and UPDRS and cognitive (MMSE and FAB) functions both preoperatively and postoperatively. We used Mann–Whitney *U* tests for comparisons between pre‐ and postoperative urodynamic parameters. The chi‐square test was used for comparison of the prevalence of detrusor overactivity preoperatively and postoperatively. Statistical significance was set at *p* < 0.05.

## 3 RESULTS

**Figure 1:**
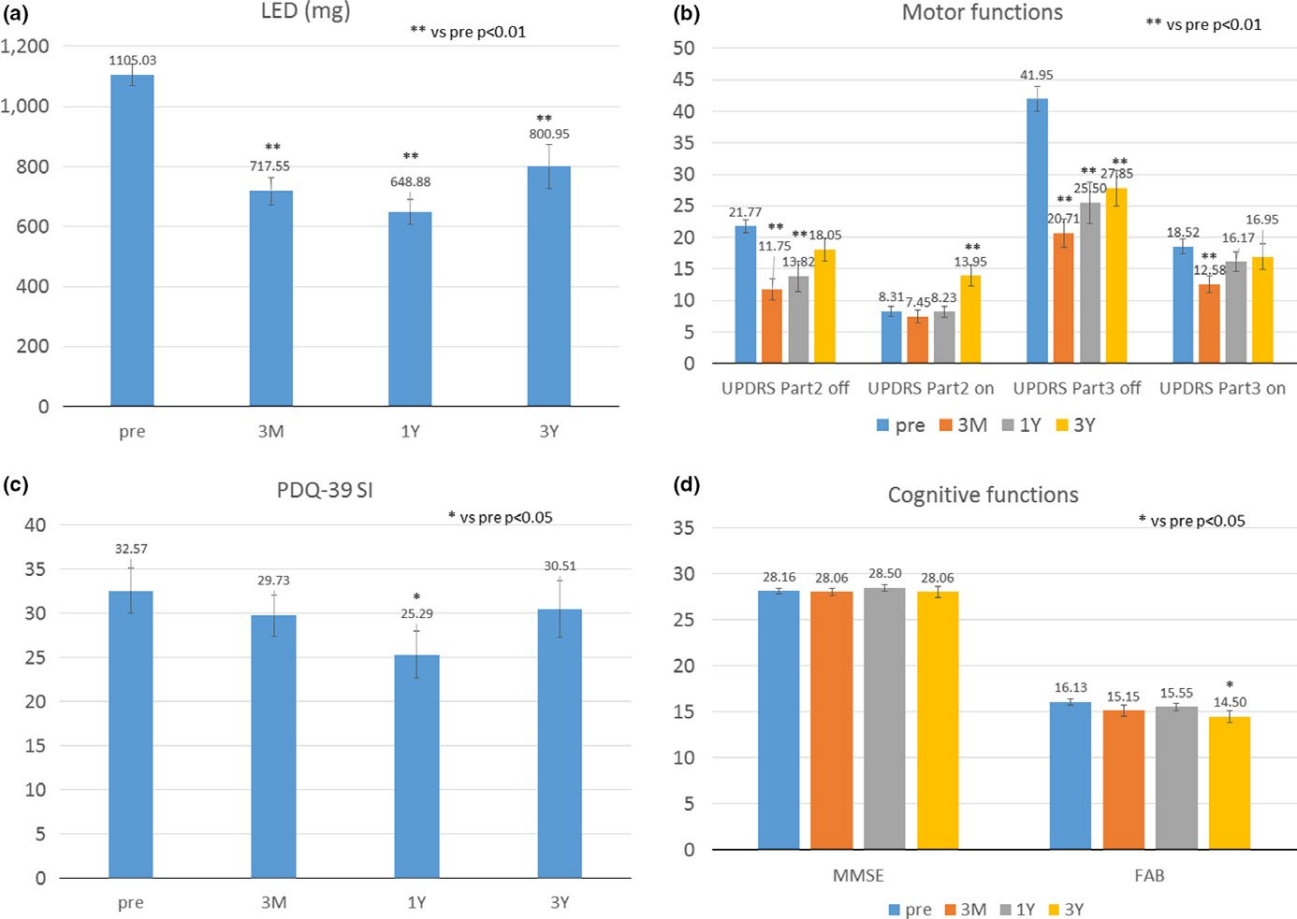
Temporal changes in levodopa equivalent dose (LED), Unified Parkinson's Disease Rating Score (UPDRS), Mini Mental State Examination (MMSE), Frontal Assessment Battery (FAB), and Parkinson's disease questionnaire‐39 (PDQ‐39) SI. LED reduced significantly at each follow‐up points after deep brain stimulation (DBS). The scores of UPDRS‐II and III during off‐phase also significantly decreased after DBS. Although the scores of MMSE did not significantly change after DBS, the score of FAB significantly decreased 3 years after DBS. The scores of PDQ‐39 SI significantly decreased 1 year after DBS

Twenty‐eight patients (mean age: 65.5 ± 1.6 years, mean disease duration: 11.8 ± 1.0 years) completed the preoperative urinary symptoms questionnaire. Of the 28 patients, 14 completed the postoperative urinary symptoms questionnaire after 3 months, 18 patients completed it after 1 year, and 10 patients completed it after 3 years. The temporal changes in LED, UPDRS, MMSE, FAB, and PDQ‐39 SI are represented in Figure 1. LED reduced significantly at each follow‐up points after DBS. The scores of UPDRS‐II and III during off‐phase also significantly decreased after DBS. Although the scores of MMSE did not significantly change after DBS, the score of FAB significantly decreased 3 years after DBS. The scores of PDQ‐39 SI significantly decreased 1 year after DBS. The troublesome dyskinesia was dramatically alleviated in PD patients who underwent GPi‐DBS.

Additionally, to treat urinary symptoms, three patients used anticholinergics both pre‐ and postoperatively. One patient used α‐blocker only preoperatively and quitted taking α blocker postoperatively due to the appearance of orthostatic hypotension. Postoperatively, three patients started to use β3 adrenoceptor agonist which was commercially available after surgery.

**Figure 2:**
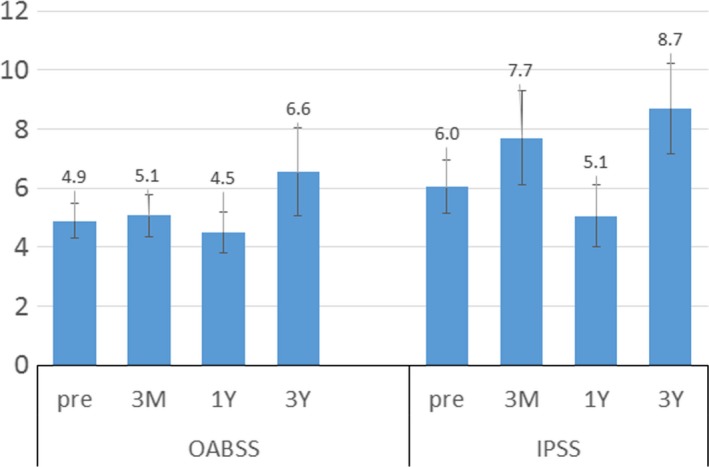
Temporal changes in the overactive bladder symptom score (OABSS) and International Prostate Symptom Score (IPSS). The OABSS and IPSS did not significantly change after deep brain stimulation (DBS)

The mean OABSS changed from 4.9 ± 0.7 at baseline to 5.1 ± 0.7 after 3 months, 4.5 ± 0.8 after 1 year, and 6.6 ± 0.7 after 3 years; however, these changes were not statistically significant (Figure 2). The mean IPSS changed from 6.0 ± 0.9 at baseline to 7.7 ± 1.6 after 3 months, 5.1 ± 1.1 after 1 year, and 8.7 ± 1.5 after 3 years; however, these changes were not statistically significant (Figure 2). The subscores of the OABSS and IPSS did not show significant changes at each follow‐up period postoperatively. Among the subscore of OABSS and IPSS, night‐time urinary frequency was the most bothersome urinary symptoms at each follow‐up points.

We also assessed the correlational coefficients between urinary symptoms and motor functions and HRQOL. The OABSS (*r* = 0.837, *p* = 0.01) and IPSS (*r* = 0.928, *p* = 0.001) showed significant positive correlations with HRQOL (PDQ‐39 SI) at 3 months postoperatively (Table 1). The OABSS and the motor functions assessed by UPDRS‐III scores during the on‐phase had significant positive correlations at 3 months postoperatively. ADL assessed by UPDRS‐II scores during the off‐phase had significant positive correlations with OABSS (*r* = 0.686, *p* = 0.02) and IPSS (*r* = 0.666, *p* = 0.036) at 3 years postoperatively (Table 1). Regarding the correlation between the subdomain of HRQOL (PDQ‐39) and urinary symptoms (IPSS and OABSS), ADL, emotional well‐being, and cognition had significant positive correlations with urinary symptoms (IPSS and OABSS) at 3 months postoperatively (Table 2).

**Table 1:** Correlations between urinary symptoms (IPSS and OABSS) and motor functions (UPDRS part II and part III), cognitive functions (MMSE and FAB), and HRQOL (PDQ‐39 SI)

	Part II on	Part II off	Part III on	Part III off	PDQ‐39 SI	MMSE	FAB
pre							
OABSS total							
*r*	0.172	−0.232	0.251	−0.216	−0.199	−0.087	−0.274
*p*	0.457	0.312	0.273	0.347	0.413	0.714	0.256
IPSS total							
*r*	−0.062	−0.044	0.268	0.021	−0.148	−0.066	−0.212
*p*	0.789	0.850	0.240	0.930	0.545	0.782	0.384
3 months							
OABSS total							
*r*	0.636	0.528	0.740*	0.641	0.837**	−0.202	−0.258
*p*	0.066	0.144	0.023	0.063	0.010	0.603	0.503
IPSS total							
*r*	0.610	0.581	0.630	0.543	0.928**	−0.272	−0.249
*p*	0.081	0.101	0.069	0.131	0.001	0.479	0.518
1 year							
OABSS total							
*r*	0.419	0.296	0.059	0.068	0.204	0.274	0.017
*p*	0.154	0.327	0.849	0.826	0.526	0.364	0.955
IPSS total							
*r*	0.352	0.262	−0.045	0.066	0.074	0.356	−0.037
*p*	0.238	0.387	0.885	0.830	0.819	0.233	0.905
3 years							
OABSS total							
*r*	0.058	0.686*	0.012	0.357	−0.025	−0.224	0.229
*p*	0.865	0.020	0.973	0.281	0.954	0.563	0.553
IPSS total							
*r*	−0.399	0.666*	−0.396	0.495	0.096	0.207	0.825*
*p*	0.253	0.036	0.257	0.145	0.820	0.623	0.012

FAB: Frontal Assessment Battery; HRQOL: health‐related quality of life; IPSS: International Prostate Symptom Score; MMSE: Mini Mental State Examination; OABSS: overactive bladder symptom score; PDQ‐39: Parkinson's disease questionnaire‐39; UPDRS: Unified Parkinson's Disease Rating Score.

*p < 0.05.

**p < 0.01.

**Table 2:** Correlation between urinary symptoms (OABSS and IPSS) and subscores of HRQOL (PDQ‐39)

	Mobility	ADL	Emotional	Stigma	Social support	Cognition	Communication	Bodily discomfort
pre								
OABSS total								
*r*	0.057	0.100	0.144	0.071	0.172	−0.130	−0.010	−0.203
*p*	0.787	0.635	0.492	0.737	0.412	0.537	0.962	0.331
IPSS total								
*r*	−0.069	0.284	0.013	−0.381	−0.091	0.160	0.210	−0.132
*p*	0.743	0.169	0.951	0.060	0.664	0.446	0.314	0.529
3 months								
OABSS total								
*r*	0.480	0.616*	0.725**	0.255	0.346	0.649*	0.436	0.180
*p*	0.114	0.025	0.005	0.401	0.247	0.016	0.137	0.557
IPSS total								
*r*	0.366	0.581*	0.773**	0.443	0.433	0.648*	0.531	0.409
*p*	0.242	0.037	0.002	0.130	0.140	0.017	0.062	0.165
1 year								
OABSS total								
*r*	−0.399	−0.014	−0.284	−0.353	0.104	−0.089	0.281	−0.146
*p*	0.177	0.963	0.347	0.236	0.736	0.772	0.352	0.635
IPSS total								
*r*	−0.512	−0.054	−0.267	−0.433	0.064	0.100	0.307	−0.190
*p*	0.074	0.862	0.377	0.140	0.837	0.744	0.308	0.533
3 years								
OABSS total								
*r*	−0.117	0.248	−0.190	−0.609	−0.443	0.088	0.012	−0.076
*p*	0.782	0.553	0.653	0.109	0.272	0.836	0.977	0.858
IPSS total								
*r*	−0.398	−0.031	0.138	−0.463	−0.274	0.152	0.106	0.178
*p*	0.329	0.941	0.744	0.248	0.512	0.720	0.803	0.673

HRQOL: health‐related quality of life; IPSS: International Prostate Symptom Score; OABSS: overactive bladder symptom score; PDQ‐39: Parkinson's disease questionnaire‐39.

*p < 0.05.

**p < 0.01.

None of the UDS parameters, such as urinary flow rate, postvoid residuals (before DBS: 28.5 ± 11.0 ml, post‐DBS: 42.0 ± 14.3 ml), first desired volume (before DBS: 134.7 ± 29.5 ml, post‐DBS: 108.2 ± 15.9 ml), and maximum bladder capacity (before DBS: 234.8 ± 35.7 ml, post‐DBS: 226.7 ± 21.5 ml), were significantly changed by DBS. Moreover, the prevalence of detrusor overactivity (before DBS: 84.6%, post‐DBS: 84.6%) and bladder contractility as assessed by Schäfer's nomogram did not change significantly after DBS (Table 3).

**Table 3:** UDS parameter

	pre‐DBS	post‐DBS	*p* value
Average flow rate (ml/s)	3.40 ± 1.03	3.14 ± 0.73	0.838
Maximum flow rate (ml/s)	8.40 ± 1.91	7.57 ± 1.42	0.730
First desired volume (ml)	134.7 ± 29.5	108.2 ± 15.9	0.481
Maximum desired volume (ml)	234.8 ± 35.7	226.7 ± 21.5	0.848
Postvoid residuals (ml)	28.5 ± 11.0	42.0 ± 14.3	0.487
Prevalence of detrusor overactivity	84.60%	84.60%	1.00

DBS: deep brain stimulation; UDS: urodynamic study.

## 4 DISCUSSION

The present study showed that DBS did not significantly improve urinary symptoms in PD patients. DBS also did not significantly change the urodynamic parameters. However, the OABSS and IPSS showed significant positive correlations with HRQOL as evaluated by the PDQ‐39, particularly at 3 months postoperatively. The OABSS and motor functions as assessed by UPDRS‐III scores during the on‐phase had significant positive correlations at 3 months postoperatively. ADL assessed by UPDRS‐II scores during the off‐phase had positive significant correlations with the OABSS and IPSS at 3 years postoperatively. Multiple subdomains of the PDQ‐39, such as ADL, emotional well‐being, cognition, had significant positive correlations with urinary symptoms 3 months after surgery. Because a higher OABSS and IPSS score represents worse urinary symptoms, the significant positive correlations between the PDQ‐39 and urinary symptoms (OABSS and IPSS) score indicated that the severity of urinary symptoms partially contributes to HRQOL.

Recent findings have suggested that non‐motor symptoms are prevalent and severe in patients with PD (Schapira et al., 2017). Although drug therapy is common for urinary symptoms in patients with PD, the responsiveness of drug therapy is not sufficient (Sakakibara et al., 2016). Therefore, DBS might be another treatment option for urinary symptoms. Some experimental and functional imaging studies have supported the idea that DBS might be suitable for ameliorating urinary dysfunctions in patients with PD. However, the effects of DBS on urinary dysfunctions are not fully efficacious and controversial in comparison to the improvement in motor complications in patients with PD (Finazzi‐Agrò et al., 2003; Herzog et al., 2006, 2008; Sakakibara et al., 2003; Seif et al., 2004; Winge et al., 2007; Witte et al., 2018).

The results of our study revealed that urinary symptoms did not significantly improve following DBS, and none of the urodynamic parameters changed postoperatively. Our UDS results reported a decreased bladder capacity and high prevalence of detrusor overactivity (84.6%), which indicates the presence of severe storage dysfunction preoperatively and postoperatively. The PVR was relatively small compared with that of multiple system atrophy, and bladder contractility assessed by Schäfer's nomogram was preserved, suggesting that voiding dysfunction was mild in this study (Yamamoto, Asahina, et al., 2017). The UDS results in the present study were compatible with those of previous studies demonstrating that storage dysfunctions are prevalent and severe in PD (Sakakibara et al., 2012; Uchiyama et al., 2011). However, urinary symptoms score (OABSS and IPSS) are relatively low compared to UDS findings. These discrepancies might be partially explained by the notion that PD patients underestimate urinary symptoms compared to motor symptoms and other several non‐motor symptoms, which might limits the interpretation of urinary symptoms score in this study. Another reason why urinary dysfunction did not significantly improve after DBS might be that the mean disease duration for patients with PD in this study was longer compared with that in previous studies, in which non‐motor symptoms were usually severe.

Importantly, the present study revealed the detailed relationships between HRQOL and urinary symptoms and their temporal changes after DBS. Our results suggest that multiple subdomains of HRQOL had significant positive correlations with urinary symptoms 3 months after surgery, suggesting that the severity of urinary symptoms partially contributes to HRQOL. It is reasonable that ADL subdomain of HRQOL had significant positive correlations with urinary symptoms, because improvement in ADL might lead to better performance in excretion activity. It is interesting that emotional well‐being also had significant positive correlations with urinary symptoms, indicating that severity of urinary symptoms might affect neuropsychiatric symptoms. Although few reports have examined the relationship between HRQOL and urinary symptoms before and after DBS, Gallagher et al reported that urinary symptoms had significant positive correlations with the PDQ‐39 SI and its multiple subdomains in patients with PD (mean disease duration: 7.8 ± 7.5 years) (Gallagher, Lees, & Schrag, 2010). It is reasonable that urinary symptoms are essentially positively correlated with HRQOL at 3 months postoperatively; however, it is also important to note that the relationship between urinary symptoms and HRQOL changed after DBS, and their relationships were heterogeneous.

Concerning the relationships between urinary symptoms and motor functions, ADL assessed by UPDRS‐II scores during the off‐phase had positive significant correlations with the OABSS and IPSS at 3 years postoperatively. These results also indicated that ADL might have significant contributions to urinary symptoms. For example, patients with better ADL are able to go to toilets before the emergence of urinary urgency, which might decrease the urinary urgency and urgent urinary incontinence.

This study had some limitations. A major limitation was that only 14, 18, and 10 of the enrolled patients completed follow‐up evaluations at 3 months, 1 year, and 3 years postoperatively, respectively. The follow‐up periods for 10 patients were <1 year after DBS and those for 18 patients were <3 years after DBS; these follow‐up periods are currently under investigation. Fourteen patients skipped the follow‐up evaluation at 3 months postoperatively due to personal reasons. It is difficult for some patients to schedule frequent admissions for follow‐up evaluations. Therefore, the small number of patients at each follow‐up period does not reflect a high drop‐out rate in this study. Another limitation was that only 13 patients agreed to participate in the UDS preoperatively and postoperatively, and the follow‐up periods of the UDS postoperatively differed among the patients. However, because a UDS is an invasive examination requiring catheter insertion into the urethra and rectum, it is difficult to conduct a UDS for all patients preoperatively and postoperatively. Furthermore, the baseline urinary symptoms‐scores are low, suggesting that PD patients in this study do not consider urinary symptoms are bothersome. However, the results of UDS indicated severe urinary storage dysfunction such as low maximal capacity and high prevalence of detrusor overactivity. These points should be examined in the future. It is also important to discuss the effect of drug therapy for urinary symptoms before and after DBS. Three patients started to use β3 adrenoceptor agonist because β3 adrenoceptor agonist was commercially available only after DBS surgery in some patients, which made the interpretation of the present result complicated. Although the effect of β3 adrenoceptor agonist on the urinary symptoms in PD is not fully understood, recent retrospective study suggested that 50% of PD patients reported improvement in OAB after administration of β3 adrenoceptor agonist (Peyronnet et al., 2018). Because only three patients started to use β3 adrenoceptor agonist after DBS, the administration of β3 adrenoceptor agonist might have only subtle effects on overall results of this study.

In addition, we could not examine the effect of difference between STN and GPi‐DBS on urinary dysfunction, because only six patients underwent GPi‐DBS.

## 5 CONCLUSIONS

Deep brain stimulation did not significantly change urinary dysfunctions in patients with PD. Urinary symptoms might partially contribute to HRQOL at 3 months postoperatively and to ADL during the off‐phase at 3 years postoperatively.
